# Ruxolitinib-based combinations in the treatment of myelofibrosis: worth looking forward to

**DOI:** 10.1007/s00277-020-04028-z

**Published:** 2020-04-24

**Authors:** Yujin Li, Shirong Zhu, Weiyi Liu, Jing Ming, Xueying Wang, Xiaomei Hu

**Affiliations:** 1 Department of Hematology, Xiyuan Hospital, China Academy of Chinese Medical Sciences, Beijing, 100091 China; 2 Graduate School, China Academy of Chinese Medical Sciences, Beijing, 100700 China; 3 Graduate School, Beijing University of Chinese Medicine, Beijing, 100029 China

**Keywords:** Myelofibrosis, Ruxolitinib, Hematology, Ruxolitinib-based combinations

## Abstract

Ruxolitinib is a targeted drug to treat myelofibrosis (MF). Ruxolitinib has significant advantages in spleen reduction and increasing 5-year overall survival (OS), and ruxolitinib-based combinations might provide more benefits than ruxolitinib monotherapy. In this review, we focus on the data of ruxolitinib-based combinations therapies and treatment-related adverse events (AEs) and safety. We analyzed and summarized the data of ruxolitinib-based combinations. Ruxolitinib combined with prednisone + thalidomide + danazol (TPD), panobinostat, pracinostat, azacytidine, or hydroxyurea has well reduced spleen. Ruxolitinib combined with danazol or TPD had well therapies in improvement of hemoglobin (Hgb) and platelets (PLT). Most ruxolitinib-based combinations therapies showed a superior benefit on reduced treatment-related AEs than ruxolitinib monotherapy. Treatment-related AEs and dose modification affect the safety and tolerability of ruxolitinib-based combinations. Genetic testing before treatment is recommended. To provide better clinical guidance, comparisons of these randomized controlled trials with the trials of ruxolitinib alone are necessary. This review suggests that the clinical application of ruxolitinib-based combinations is worth waiting for.

## Introduction

Myelofibrosis (MF) is a breakpoint cluster region protein (BCR)-Abelson tyrosine-protein kinase (ABL)–negative myeloproliferative neoplasm (MPN) and represents a group of tumors caused by abnormal proliferation of one or more myeloid cells. MPN includes polycythemia vera (PV), essential thrombocythemia (ET), and MF [1, 2]. During disease progression, patients with MF might have symptoms of progressive anemia, thrombosis, splenomegaly, extramedullary hematopoiesis, myelofibrosis, fatigue, crippling constitutional symptoms (night sweats, fever, and weight loss), bone pain, and pruritus [3]. Traditional treatments are mainly used to relieve symptoms and prevent thrombosis. In 2005, the Janus kinase (JAK)2/V617F gene mutation was identified in the DNA of patients with MPN [4]. Follow-up studies confirmed that *JAK2* gene mutation was important in the diagnosis and treatment of these patients. In 2011, ruxolitinib was approved as a potent inhibitor of *JAK1/2* for the treatment of patients with MF with the International Prognostic Scoring System (IPSS) intermediate risk–2/high-risk [5, 6]. In addition to *JAK2*, mutations of *MPL* (encoding myeloproliferative leukemia protein) and *CALR* (encoding calreticulin) are also common [7, 8].

Ruxolitinib has significant advantages in spleen reduction and increasing 5-year OS [9–12]; however, it is often accompanied by treatment-related adverse events (AEs), such as infections and cytoreduction [13–16]. Numerous studies have identified safety problems when using ruxolitinib alone. These problems are mainly divided into hematological and non-hematological AEs. Hematological AEs mainly include anemia and thrombocytopenia, and non-hematological AEs include headache, dizziness, and bronchitis [9, 12, 17–19]. These AEs represent a challenge to clinical medicine strategy making and also reduce the quality of life of patients. Other JAK inhibitors have been studied; however, because of their corresponding toxicities, it is hard for them to exceed or replicate the efficacy of ruxolitinib in the short term [20, 21]. Ruxolitinib-based combinations that maintain the efficacy of ruxolitinib and reduce the impact of AEs have aroused interest. To improve the efficacy of ruxolitinib and to address the unmet clinical needs, a few combination approaches have been tested in MF [22].

## Ruxolitinib combined with danazol could significantly improve PLT levels and anemia

Anemia is a common manifestation of MF. Ruxolitinib can aggravate cytopenia, which becomes a factor in worsening the disease. Ruxolitinib dose reduction or discontinuation to offset or reduce the associated cytopenia is used clinically. In this case, some patients would benefit less or lose the opportunity to receive ruxolitinib treatment.

The mechanism of danazol in the treatment of anemia is not yet clear. Previous studies on MF-related anemia showed that the use of danazol alone or combined with other drugs could improve hemoglobin levels [23, 24]. Danazol could significantly improve platelet (PLT) levels and anemia (without transfusion dependency) [25]. Thus, ruxolitinib combined with danazol has become a new and feasible treatment.

The trial results of ruxolitinib combined with danazol showed that 31% of patients (in whom anemia could be assessed) had increased hemoglobin by more than 1.0 g/dL (Table 1). Of the 9 patients with prior JAK inhibitor exposure, 5 patients (55.5%) and 8 patients (88.9%) had stable or increasing Hgb levels and PLT levels, respectively. According to the criteria of the International Working Group for Myelofibrosis Research and Treatment (IWG-MRT), stable disease (SD), clinical improvement (CI), partial response (PR), and progressive disease (PD) were 64%, 21%, 8%, and 8%, respectively [26].

**Table 1:** Baseline characteristics of patients

NCT number	Combination drug	Enrollment (*N*)	Median age (years)	Risk stratification, *N*(%)	Ruxolitinib dose	Ruxolitinib-naïve, *N*(%)	Combination drug dose	Driver mutation status, *N* (%)	Reference
NCT01732445	Danazol	*N* = 14	70.5 (43–78)	DIPSS: Int-1: *N* = 3(21) Int-2: *N* = 7(50) High: *N* = 4(29)	5 or 10 mg/bid	*N* = 9(65)	200 mg/tid	JAK2: *N* = 6(43) Other mutational analysis was not performed	[26]
--	Thalidomide Prednisone Danazol	*N* = 7	55 (36–58)	DIPSS: Int-1: *N* = 2(29) Int-2: *N* = 4(57) High: *N* = 1(14)	10 mg/day: *N* = 2 30 mg/day: *N* = 3 40 mg/day: *N* = 2	*N* = 4(57)	--	JAK2: *N* = 4(57) CALR: *N* = 1(14) MPL: *N* = 1(14) Triple-negative: *N* = 1(14)	[27]
NCT01375140	Lenalidomide	*N* = 31	66 (37–82)	DIPSS: Int-1: *N* = 14(45) Int-2: *N* = 11(35) High: *N* = 6(19)	15 mg/bid, 1–28 days	*N* = 31(100)	5 mg/day, 1–21 days	JAK2: *N* = 26(84)	[28]
--	Panobinostat	*N* = 15	64 (46–78)	DIPSS: Int-1: *N* = 1(7) Int-2: *N* = 14(93)	10–15 mg/bid	*N* = 10(67)	10–20 mg tiw/qow	JAK2: *N* = 10(67) CALR: *N* = 3(20) MPL: *N* = 1(7)	[29]
NCT02267278	Pracinostat	*N* = 20	66 (56–78)	IPSS: Int-1: N = 3(15) Int-2: *N* = 6(30) High: *N* = 11(55)	5 or 15 mg/bid	--	60 mg every other day for 3 out of every 4 weeks	JAK2: *N* = 17(85) CALR: *N* = 1(5) MPL: *N* = 1(5) Triple-negative: *N* = 1(5)	[30]
--	Azacytidine	*N* = 41	66 (48–87)	--	15 or 20 mg/bid	--	25–75 mg/m^2^	JAK2: *N* = 23(56)	[31]
NCT01787487	Azacytidine	*N* = 46	66 (48–87)	DIPSS: Int-1: *N* = 16(35) Int-2: *N* = 20(43) High: *N* = 10(22)	5, 15, or 20 mg/bid	*N* = 46(100)	25, 50, or 75 mg/m^2^	JAK2: *N* = 24(52) CALR: *N* = 5(17) MPL: *N* = 2(7)	[32]
--	Hydroxyurea	*N* = 53	67 (54–82)	DIPSS: Int-1: *N* = 8(15) Int-2: *N* = 25(47) High: *N* = 20(38)	5 mg/bid: *N* = 2 10 mg/bid: *N* = 12 15 mg/bid: *N* = 20 20 mg/bid: *N* = 19	–	Median daily dose: 1.500 mg/day (range, 500–2000)	JAK2: *N* = 37(70) CALR: *N* = 6(11) MPL: *N* = 2(4) Triple-negative: *N* = 8(15)	[33]
--	ESAs	*N* = 59	69 (48–81)	DIPSS: Int-1: *N* = 4(7) Int-2: *N* = 42(71) High: *N* = 13(22)	5 mg/tid	--	Epoetin alpha/beta/zeta (weekly): 40,000 iu/30,000 iu/159 μg	JAK2: *N* = 42/50(84) CALR: *N* = 7/50(14) MPL: *N* = 1/50(2)	[34]
--	HSCT	*N* = 22	59 (42–76)	IPSS: Int-1: *N* = 3(14) Int-2: *N* = 14(64) High: *N* = 5(23)	5 mg/bid: *N* = 5 15 mg/bid: *N* = 5 20 mg/bid: *N* = 12	--	--	JAK2 positive: *N* = 15(68) JAK2 negative: *N* = 7(32)	[35]
--	HSCT	*N* = 100	59 (32–72)	DIPSS: Int-1: *N* = 40(40) Int-2: *N* = 48(48) High: *N* = 6(6) Not available: *N* = 6(6)	–	--	--	JAK2 positive: *N* = 62(62) JAK2 negative: *N* = 37(37) Not available: *N* = 1(1)	[36]
--	HSCT	*N* = 14	58 (33–68)	IPSS: Int-1: *N* = 2(14) Int-2: *N* = 6(43) High: *N* = 6(43)	5, 10, 15, 20 mg/bid	--	--	JAK2 positive: *N* = 9(64)	[37]

*DIPSS*, Dynamic International Prognostic Scoring System; *IPSS*, International Prognostic Scoring System; *DFS*, disease-free survival

Danazol has been used to treat anemia in patients with MF for a long time. Treatment with ruxolitinib combined with danazol improved hematological and non-hematological AEs; however, the number of patients (*n* = 14) enrolled in the trial was small, such that the results lack sufficient data support. After the inclusion of patients, there was no complete gene sequencing, such as that for *CALR* and *MPL* gene mutations; therefore, an in-depth analysis of the efficacy mechanism could not be conducted. The observation period was too short to draw a definitive conclusion and requires further research, because danazol’s response time is generally 3–6 months, and its benefits may have been underestimated [25].

## Ruxolitinib combined with immunomodulatory agents

MF is regarded as a chronic inflammation-related disease [38, 39]. Immunomodulatory agents have an established role in the treatment of myelofibrosis and demonstrate pleiotropic activities, including anti-angiogenesis, anti-tumor, regulation of cellular immunity, inhibition of NF-κB, apoptosis, and selective inhibition of pro-inflammatory cytokines [40]. Commonly used immunomodulatory agents include thalidomide, lenalidomide, and pomalidomide. As second-generation immunomodulator drugs, lenalidomide and pomalidomide show stronger immunomodulatory effects and angiogenesis inhibition, and improved safety, compared with thalidomide [41].

In recent years, thalidomide, lenalidomide, and pomalidomide have induced an adverse reaction rate of about 20–40% [42]. Anemia and thrombocytopenia are the most common ruxolitinib treatment-related AEs; however, research has demonstrated that the efficacy of thalidomide or lenalidomide monotherapy are not ideal [43].

In the COMFORT-I and COMFORT-II trials, the dose reduction and discontinued treatment because of anemia were 6% and 5%, respectively [12, 44]. Early trials had shown that thalidomide in low doses (< 100 mg/day) could improve symptoms such as anemia, thrombocytopenia, and splenomegaly [45, 46]. In a retrospective cohort study, ruxolitinib combined with low dose thalidomide, stanozolol, and prednisone significantly modulated initial hematological toxicity and improved anemia [47]. Although the use of ruxolitinib combined with immunomodulatory agents seems complicated, it is theoretically feasible [48].

### Ruxolitinib combined with PTD showed excellent tolerability and safety

From the trial results of ruxolitinib combined with PTD (Table 1), five of the seven patients had varying degrees of anemia before treatment. After receiving treatment with ruxolitinib and PTD, that five achieved CI, and two patients had SD. The Hgb concentration and PLT counts of the three patients had decreased when receiving ruxolitinib monotherapy, which then increased significantly after combination therapy. No hematological AEs and grade III/IV non-hematological AEs occurred in any of the patients [27].

Low dose thalidomide combined with prednisone had demonstrated improvements in anemia of about 50%, and the tolerance was better than that of traditional dose thalidomide [49, 50]. Therefore, the ruxolitinib combined with PTD could not only improve anemia and PLT counts, but also had better safety.

### For ruxolitinib combined with lenalidomide, the dose is the key, and ruxolitinib plays a more vital role in the treatment

In a trial of lenalidomide combined with ruxolitinib (Table 1) [28], MF patients were given 15 mg/bid ruxolitinib for twenty-eight consecutive days, plus 5 mg/day of lenalidomide on days 1–21. Only 35% of patients did not require a dose interruption in the first 3 months. About 74% of patients required a dose interruption, with or without a dose decrease, because of toxicity, and 19% of patients were given additional one or two drugs because of a lack of satisfactory efficacy. In addition, 45% of patients completely discontinued lenalidomide within 3 months of initiation. The trial was terminated early because of the possible association with myelosuppression. In this study, 55% of patients achieved an IWG-MRT-defined spleen response, and all achieved CI in the palpable spleen size reduction, including 100% spleen reduction in seven patients; however, none of the patients had a measurable CI in the PLT count.

This study failed to meet the predetermined treatment effect, and the combination induced severe myelosuppression. Concomitant initiation and continuation of both drugs was difficult because of toxicity, with most discontinuations occurring early when the hematological toxicities were at their peak. Therefore, dose interruption, modification, and discontinuation should be verified in further trials to gradually improve the safety and tolerability. Studies on immunotherapy for patients with MF are very limited, and their mechanism remains to be defined [51]. Immunotherapeutic approaches are expanding and changing, which will extend the therapeutic armamentarium for patients with myeloproliferative neoplasms.

## Ruxolitinib combined with a histone deacetylase inhibitor

### Ruxolitinib combined with panobinostat has well tolerated and reduced the spleen size

Panobinostat is a novel pan-histone deacetylase inhibitor (HDACi). HDACis use multiple mechanisms to kill bone marrow cancer cells and can disrupt the interaction between *JAK2* and heat shock protein 90 (HSP90), which specifically enhances acetylation of histone H3, H4, and HSP90. Long-term low dose panobinostat (15 mg three times a week) could improve anemia, myelofibrosis, splenomegaly, and leukocytosis in patients with MF who have received panobinostat alone [52, 53]. According to pre-clinical trials, panobinostat inhibits the expression of JAK2/V617 gene, and in combination with *JAK2* selective kinase inhibitors, it inhibits JAK/STAT signaling more strongly than either drug alone [54].

Panobinostat demonstrated an advantage in spleen reduction and improvement in bone marrow (BM) fibrosis, and the trial results of ruxolitinib combined with panobinostat showed synergistic activity in pre-clinical MF models [55]. When administered at lower doses over a prolonged duration, panobinostat was more effective and better tolerated in the treatment of patients with MF.

In the results of ruxolitinib combined with panobinostat, ruxolitinib was administered at 10–15 mg/bid and panobinostat at 10–20 mg tiw/qow (Table 1). Jak2 gene mutation was presented in 10/15 (67%) patients. According to IWG-MRT, 6 (40%) patients achieved CI and 8 (53%) patients attained SD. Five of fifteen (33%) patients achieved either a 35% spleen volume response or a 50% reduction in spleen size (by palpation). The most common treatment-related AE was anemia which occurred in 7 patients (47%) at the end of 6 cycles; 6 of 7 patients were grade 3/4 anemia [29].

In another trial of ruxolitinib combined with panobinostat showed a tolerable safety profile, with few dose-limiting toxicities and acceptable rates of grade 3/4 anemia (34.2%) and thrombocytopenia (21.2%) [56]. The result of spleen reduction and grade 3/4 AEs were similar to those of patients who received single agent ruxolitinib in the phase III COMFORT-I and COMFORT-II trials [57]. In another study, 57% and 39% of patients achieved spleen responses at week 24 and 48, respectively. Some patients had JAK2/V617F allele burden reduction and improved BM fibrosis, and AE rates were consistent with ruxolitinib and panobinostat monotherapy treatment [58]. Treatment with ruxolitinib combined with panobinostat was well tolerated and reduced the spleen size.

### Ruxolitinib combined with pracinostat showed non-ideal efficacy and tolerance

Pracinostat, a pan-HDACi, is a potent oral inhibitor of class 1, 2, and 4 histone deacetylases (HDACs), which showed modest single agent efficacy in myelofibrosis [59, 60]. In the study of ruxolitinib combined with pracinostat (Table 1), ruxolitinib was received alone for 3 cycles, and pracinostat was added in cycle 4. A total of 25 patients with MF were enrolled, of whom 20 received both drugs. According to the IWG-MRT 2013 criteria, 80% of patients had objective responses (all “CI”). The rate of spleen response (by palpation) was 74%. According to the National Cancer Institute Common Terminology Criteria for AEs, anemia and thrombocytopenia were the most common. Six patients discontinued pracinostat because of AEs [30].

For ruxolitinib combined with pracinostat, the efficacy and tolerance were not ideal. In this study, deterioration of anemia requiring transfusion was a major reason for the poor tolerance of the combination. The method of drug delivery made it difficult to evaluate efficacy accurately. Most responses occurred before pracinostat initiation. Therefore, whether ruxolitinib combined with pracinostat has clinical sustainability requires further optimized, larger sample size studies.

## Ruxolitinib combined with azacytidine has potential synergy for spleen length reduction and BM fibrosis improvement

Azacytidine (AZA) is a DNA methyltransferase (DNMT) inhibitor. In 2002, AZA became the first US Food and Drug Administration (FDA)–approved treatment for myelodysplastic syndromes (MDS). AZA has demonstrated significant efficacy in the treatment of MDS, in which it not only prolongs median survival and improves the quality of life, but also reduces the conversion of MDS to acute myeloid leukemia (AML) [31, 61, 62]. Medical researchers hypothesized that the combination of AZA and ruxolitinib would target distinct clinical and pathological manifestations of MF, resulting in synergistic efficacy.

In one study, patients received ruxolitinib alone for the first 3 months, followed by the addition of AZA. In total, 41 patients were enrolled (Table 1). According to IWG-MRT, 27 (69%) patients had an objective response, but 10 (26%) patients had no IWG response, and 2 (5%) patients showed progression to AML on therapy. Twenty-three (79%) patients achieved > 50% palpable spleen reduction at any time on the study, which was superior to that of single agent ruxolitinib, and 26% of the spleen reductions occurred after the addition of AZA [32]. In 13 responders, a 21% median reduction in *JAK2* allele burden was achieved, and 11 BM fibrosis responders experienced a reduction in European myelofibrosis network fibrosis score. The spleen length reduction was superior to that induced by single agent ruxolitinib.

Another trial of ruxolitinib combined with AZA enrolled 46 patients. After receiving ruxolitinib treatment for 3 months, 41 of them received AZA. Twenty-four patients (71%) achieved a > 50% reduction in palpable spleen length at any time on the study. At 24 weeks, 13 patients had a reduction in *JAK2* allele burden. Of the 31 patients with sequential BM evaluations, 57% showed improvement in BM reticulin fibrosis at 24 months. The most common hematological events were anemia (72%) and thrombocytopenia (63%), and grade ≥ 3 AEs occurred in 31 patients (67%). Median hemoglobin declined to a nadir at the point of 9.4 g/dL at 12 weeks, then increased to a new steady state of about 10 g/dL at week 24 and remains above this level. Seventeen patients died within 22 months of median follow-up, and three patients died when treated with AZA combined with ruxolitinib. The mean treatment time was 18 months, and the overall discontinuation rate was 72%; however, treatment-related discontinuation rates were 8% [63].

In this study, ruxolitinib combined with azacytidine was safe and effective. About 45% of patients observed improvements in abnormal collagen or osteosclerosis. The proportion showing a spleen response was better than that in trials using a single dose of ruxolitinib [9, 44]. Reasonable dose modifications and sequential administration strategies may mitigate the cytopenias and discontinuation rates. Although the discontinuation rate of the combination was approximately consistent with that of single agent ruxolitinib, improvement in the OS, reduction of the discontinuation rate, and AEs remain a problem that requires further clinical study.

## Ruxolitinib combined with hydroxyurea is feasible in real-world practice

Hydroxyurea is an anti-neoplastic drug and an inhibitor of ribonucleotide reductase [64]. Hydroxyurea was the first choice therapy in patients with ET before ruxolitinib was approved [65]. Hydroxyurea acts by inhibiting the proliferation of BM megakaryocytes and then reducing the PLT count. Unless proved otherwise, patients who received treatment of hydroxyurea who suffered anemia along with thrombocytosis should probably be considered as suffering from disease progression [66].

In a trial, 53 patients with MF received treatment comprising ruxolitinib combined with hydroxyurea (Table 1). After combination therapy, 45% of patients had a reduction of the baseline splenomegaly, and 23% of patients had various grades of anemia and/or thrombocytopenia. At 48 weeks, the rate of spleen response increased to a maximum of 45%, and the leucocyte and PLT counts decreased significantly. The median time to discontinuation was 2.5 months. Seventeen patients died, among whom four patients had acute myeloid leukemia, and two patients had thrombotic disorders [33].

Hydroxyurea is commonly used to control thrombocytosis and leukocytosis. When hyperleukocytosis and/or increase PLT count occur during treatment with ruxolitinib alone, the combination with cytoreductive drugs was necessary. The efficacy and safety of ruxolitinib combined with hydroxyurea were validated in this trial, and when ruxolitinib monotherapy proves non-ideal, this drug combination may be regard as a useful option in patients with MF.

## Ruxolitinib combined with erythropoiesis-stimulating agents seem effective in improving anemia, and the endogenous erythropoietin levels is a good predictor of AR

Erythropoiesis-stimulating agents (ESAs) have been routinely used in treating anemia, and the combination of it with ruxolitinib has been used in the studies of preventing anemia. In the study of COMFORT-I and COMFORT-II, anemia is one of the most frequent AEs, but it is generally controllable. There were only two patients (among one hundred and forty-six patients) discontinued after 3 years in COMFORT-II trial due to their severe anemia [67]. Both COMFORT-I and COMFORT-II trials showed that anemia occurred in 8th to 12th weeks and improved at the 24th week. There was no need of transfusion or changing the dosage of the ruxolitinib during this period [12, 44]. Considering the fact that ESAs activate the JAK pathway and may potentially lead to an increase of spleen size and affect the effectiveness of the spleen response, the use of ESAs are prohibited in COMFORT-II trials (although it is not prohibited) [68, 69].

Crisà et al. [34] retrospectively evaluated fifty-nine patients who received ruxolitinib combined with ESAs for anemia and had found that the rate of anemia may be related to the combination of ruxolitinib and ESAs, and lower erythropoietin (EPO) (< 125u/I) level was an obvious predictor of anemia response. Especially, there was no significant negative impact of the ESAs on response to ruxolitinib. There was no thrombotic event in this trial, and the overall survival of patients was 4 years, which is similar to what in the COMFORT trial (Table 1).

ESAs are the major regulator of erythropoiesis. When JAK2 signaling is inhibited in patients who received the treatment with ruxolitinib, it may lead to impaired erythropoiesis and anemia. Ruxolitinib combined with ESAs can be a tool to help patients in improving anemia which is caused by JAK inhibitors. The reason why it improves anemia is probably because that the half-life of ESAs is longer than of ruxolitinib’s, and JAK2 is maybe not completely inhibited by therapeutic concentrations of ruxolitinib. According to the analysis of current clinical studies, it is still not possible to make a clear recommendation for anemia in patients treated with ruxolitinib and ESAs, but the combination is safe and does not affect the treatment of ruxolitinib [17].

## Ruxolitinib prior to allogeneic hematopoietic stem cell transplantation can improve the rate of success transplantation and spleen size reduction

Hematopoietic stem cell transplantation (HSCT) is widely used as a method to cure MF in recent years. It is recommended for MF patients to receive HSCT after having a suitable donor as soon as possible, who with the intermediate risk-2 and high-risk classified by Dynamic International Prognostic Scoring System (DIPSS), DIPSS-plus, and IPSS, and had a life expectancy of < 5 years [70, 71]. MF patients with JAK2, CALR, and MPL mutations had better disease-free survival and overall survival after transplantation than those without a mutation. Among them, CALR mutations had the best prognosis [72–74]. The spleen size and systemic symptoms of the patients before transplantation can interfere with the outcome of the transplant [75].

Stübig et al.’s study included twenty-two MF patients, including thirteen patients with primary myelofibrosis, nine patients with post-ET/PV, fifteen patients with JAK2V617F mutations, and the seven were negative (Table 1) [35]. Twenty-one patients had splenomegaly before treatment with ruxolitinib, and nineteen patients received treatment with ruxolitinib before transplantation. Three patients received treatment with ruxolitinib before second transplantation, and the median time to receive pre-transplant treatment was ninety-seven days, 86% of patients had improvement of constitutional symptoms at the time of transplantation, 41% of the patients had spleen response, and 14% had a spleen size reduction of < 50%. During follow-up, that four patients died (one was secondary myeloid acute leukemia; the other three were treatment-related mortality); the 1-year overall survival rate and disease-free survival was 81% and 76%, respectively.

Shanavas et al. showed in a retrospective multicenter study that one hundred patients had a high (61%) 2-year overall survival, and the 2-year overall survival was 91% for twenty-three patients with CI [36]. Prior treatment with JAK1/2 inhibitors did not adversely affect early outcome in post-transplantation patients with MF, and administering ruxolitinib before HSCT reduced the risk of “rebound” and “withdrawal symptoms” (Table 1).

Jaekel et al. observed that thirteen of fourteen MF patients’ occurred engraftment rate was 93% after receiving ruxolitinib, and MF-related symptoms amelioration were 71.4% [37]. Median follow-up was 9 months. Survival and treatment-related mortality rate was 78.6% and 7%, respectively (Table 1).

In the era of JAK inhibitor, HSCT remains a highly relevant treatment option for MF [76]. Spleen reduction can affect the results of HSCT [77], and the effect of ruxolitinib in spleen reduction is outstanding. Therefore, continuous ruxolitinib administration before transplantation would have a certain influence on HSCT and survival after transplantation. Prior treatment with JAK1/2 inhibitors did not adversely impact early post-transplantation outcomes in MF, and the included patients without spleen reaction may be associated with genetic mutations. Since ruxolitinib is a JAK1/2 inhibitor, it can also cause severe inflammation while improving the symptoms of patients, and standardized clinical tests or combined with anti-infective treatments can benefit patients (Table 2).

**Table 2:** Treatment data of ruxolitinib-based combinations

	Median Hgb	Median Hgb	Median PLT	Median PLT	Transfusion dependence *N*(%)	Transfusion dependence *N*(%)	AR *N*(%)	≥ 3 grade Hematologic AEs *N*(%)	Survival	BM fibrosis	Symptoms response	IWG-MRT response criteria	Spleen reduction *N*(%)	AML *N*(%)	Median follow-up (months)	Reference
Combination drug	Start ruxolitinib	After combination	Start ruxolitinib	After combination	Start ruxolitinib	After combination										
Danazol	9.0 g/dL (8.3–12.4)	Maximum change > 1.0 g/dL: *N* = 5(36)	157 × 10^9^/L (54–441)	Maximum change > 50 × 10^9^/L: *N* = 5(36)	*N* = 5(36)	--	*N* = 13/14(93)	*N* = 10(71)	--	--	MPN-TSS: Improvement ≥ 50% *N* = 4	SD: *N* = 9(64); CI: *N* = 3(21); PR: *N* = 1(7); PD: *N* = 1(7)	Spleen responses: *N* = 12(86)	--	--	[26]
Thalidomide Prednisone Danazol	8.7g/dL (5.7–14.4)	Increase: 3 g/dL (1.8–5.4)	123 × 10^9^/L (74–500)	Increase: 116 × 10^9^/L (13–369)	*N* = 2(29)	*N* = 0	--	*N* = 0(0)	--	--	Median MPN-SAF-TSS decrease: 16 score	--	Spleen responses: *N* = 6(86)	--	--	[27]
Lenalidomide	11 g/dL (8.9–17.5)	–	250 × 10^9^/L (27–1898)	--	--	--	--	*N* = 16	--	Reduction: *N* = 2/17(17) MF-2 to MF-1: *N* = 1 MF-2 to MF-0: *N* = 1	--	--	100% spleen reduction: *N* = 7(23) 50% spleen reduction-: *N* = 10(32)	--	28 (12–35+)	[28]
Panobinostat	9.8g/dL (8.3–12.8)	--	347 × 10^9^/L (95–672)	--	*N* = 2(13)	--	--	*N* = 6(43)	--	Reduction: *N* = 7 (53) MF-3 to MF-2: *N* = 5 (33) MF-3 to MF-1: *N* = 1 (7) MF-2 to MF-1: *N* = 1 (7)	MPN-SAF-TSS response: *N* = 3/12(25)	SD: *N* = 8 (57); CI: *N* = 6 (43)	35% spleen volume response: *N* = 5(46)	--	--	[29]
Pracinostat	10.9 g/dL (7.4–16.2)	--	253 × 10^9^/L (107–698)	--	--	*N* = 4(20)	--	*N* = 12 (60)	Median OS: 33.8 months	MF-3 to MF-1: *N* = 2 MF-3 to MF-2: *N* = 1	MPN-SAF TSS response: *N* = 12/15(80)	*N* = 5	Spleen response (by palpation): *N* = 14(74)	*N* = 1(5)	21.4 (12.5–39.1)	[30]
Azacytidine	10.1 g/dL (6.8–16.2)	--	271 × 10^9^/L (126–835)	--	--	--	--	New onset anemia: *N* = 25(64); Thrombocytopenia: *N* = 11(28)	Median OS: 38.7+ months	EUMNET fibrosis score reduction: *N* = 11(28)	--	*N* = 27/39(69) CI: *N* = 25(64); PR: *N* = 2(5)	> 50% palpable spleen reduction: *N* = 23(56)	*N* = 4(10)	20.4+ (0.5–37.3+)	[31]
Azacytidine	10.1 g/dL (6.8–16.2)	--	271 × 10^9^/L (125–1070)	--	*N* = 5(11)	*N* = 15(33)	*N* = 4/41(10)	--	DIPSS-high: Median OS: 28 (1–62) months	BM reticulin fibrosis improved: *N* = 8/14(57) BM collagen improved: *N* = 7/14(50)	MPN-SAF TSS: *N* = 25/46(54)	*N* = 7/33(21)	Spleen response: *N* = 21(62)	*N* = 6(13)	22 (5–50)	[32]
Hydroxyurea	10 g/dL (6.7–16.4)	12 weeks: 9.5 g/dL (6.8–14.6) 24 weeks: 10.3 g/dL (7–13) 48 weeks: 11.4 g/dL (7.8–14)	186 × 10^9^/L (60–1110)	12 weeks: 113 × 10^9^/L (30–652) 24 weeks: 135 × 10^9^/L (35–545) 48 weeks: 158 × 10^9^/L (78–523)	--	--	--	*N* = 7(12)	--	--	--	48 weeks: 75% patients achieved a symptoms response	Spleen responses: 12 weeks: 14/50(28) 24 weeks: 15/40(38) 48 weeks: 15/33(45)	*N* = 4(8)	28 (4–50+)	[33]
ESAs	8.7 g/dL(6–10)	--	--	--	*N* = 31(59)	--	*N* = 32/59(54)	--	2 years OS: 78% 4 years OS: 62%	--	--	--	--	--	48	[34]
HSCT	--	--	--	--	--	--	--	--	1 year OS: 81% 1 year DFS:76%	--	--	--	Before HSCT Spleen size response: 45%	*N* = 1(5)	12 (6–13)	[35]
HSCT	--	--	--	--	--	--	--	During JAK1/2 inhibitor therapy: *N* = 49(49)	2 years OS: 55%	--	--	--	--	--	After HSCT: 17 (3–53)	[36]
HSCT	11.1 g/dL(8.4–16.4)	--	--	--	--	--	--	--	1 year OS: 50%	--	--	--	--	*N* = 2(14)	After HSCT: 9 (4–43)	[37]

*Hgb*, hemoglobin; *PLT*, platelet; *AR*, anemia response; *SD*, stable disease; *CI*, clinical improvement; *PR*, partial response; *MPN-SAF TSS*, MPN Symptom Assessment Form Total Symptom Score; *EUMNET*, the European Myelofibrosis Network

## Continuously updated combination trials are expected

At present, a number of trials have been launched for ruxolitinib-based combinations, with variable results. According to the different stages of the disease and different clinical manifestations, developing the best treatment plan has become the driving force in clinical research. The ongoing trials are shown in Table 3.

**Table 3:** Current clinical trials involving ruxolitinib combinations

Drug 1	Drug 2	Phase	NCT number
Ruxolitinib	Decitabine	I/II	NCT02076191
	PRM-151	II	NCT01981850
	GS-6624	II	NCT01369498
	LDE225	I/II	NCT01787552
	PU-H71		NCT03935555
	Navitoclax	II	NCT03222609
	Umbralisib	I	NCT02493530
	Parsaclisib	II	NCT02718300
	PIM447	Ib	NCT02370706
	Itacitinib	II	NCT03144687
	Pevonedistat	I	NCT03386214
	Luspatercept	II	NCT03194542
	Peg-interferonalpha-2a	I/II	NCT02742324
	Sotatercept		
	vismodegib	Ib	NCT02593760

PRM-151 is a recombinant human pentraxin-2 molecule that can reverse cell fibrosis. Observations of 18 patients with MF who had received treatment comprising ruxolitinib combined with PRM-151 for a median of 31 months showed that the two-drug combination was well tolerated. An overall improvement in BM reticulin and collagen fibrosis grade, as well as reductions in symptoms (MPN-SAF TSS) and palpable splenomegaly were noted [78]. In another trial, 13 of 27 patients (with or without ruxolitinib) have completed at least 72 weeks. PRM-151 treatment was well tolerated, and improvements in Hgb, PLT, symptoms, and spleen appeared to increase with longer treatment duration [79]. PRM-151 produced sustained improvements in myelofibrosis-related cytopenia in some patients, and further data on this drug are eagerly awaited [80]. PRM-151 has shown promise in this regard [81].

Sonidegib is an oral selective smoothened (SMO) small molecule inhibitor that inhibits hedgehog signaling [82, 83]. In a trial of sonidegib combined with ruxolitinib, 20 of 27 patients continued to receive treatment at data cutoff, and 5 patients discontinued treatment due to AEs [84]. The most common AE was anemia (52%). The best response of ≥ 50% reduction in spleen length at any time on treatment was achieved by 25 patients (92.6%), and 15 patients (55.6%) had a non-palpable spleen. In a study of dose escalation, sonidegib combined with ruxolitinib was generally well tolerated. From the current results, sonidegib combined with ruxolitinib should be superior to either single drug.

Umbralisib (TGR-1202) is a PI3Kδ inhibitor that inhibits the PI3K/AKT signaling pathway; interestingly, PI3Kδ is highly expressed in patients with MF. Inhibition of PI3K/AKT signaling could reduce proliferation and clonal potential in *JAK2* mutant cells. In a previous trial, 11 patients were observed, among which 9 patients were evaluable, 7 patients were stable, and 8 patients showed relief of MF symptoms [85]. Another trial has proven that ruxolitinib combined with umbralisib was well tolerated. The combination of the two drugs can increase Hgb and improve spleen size [86].

Vismodegib is a Hedgehog pathway inhibitor (HPI) that binds to and inhibits SMO. Vismodegib is approved for the treatment of locally advanced and metastatic basal cell carcinoma [87]. Pre-clinical and clinical data suggest that the addition of an HPI to ruxolitinib might improve the response. In a study of vismodegib combined with ruxolitinib in patients with MF with intermediate or high-risk disease, 10 patients were enrolled and 8 patients had completed 48 weeks. The most common AEs were muscle spasm. Vismodegib combined with ruxolitinib was tolerable; however, there was no evidence that the combination therapy could improve efficacy [88].

## Discussion

Ruxolitinib has changed the pattern of MF treatment and brought new treatment directions to the clinic. However, patients with PLT < 50 × 10^9^/L or anemia, who are heavily dependent on blood transfusion, are generally not suitable for treatment with ruxolitinib, which remains an unmet need [89]. Undeniably, ruxolitinib presents new challenges to clinicians. Prevention of thrombogenesis and improvement of anemia are the main treatment targets for patients with MF. We not only hope that patients will benefit from the reduction of spleen size or improvement of symptoms after receiving treatment with ruxolitinib, but also hope that they achieve higher quality of life and longer life.

When treating patients with MF, cytopenias remain a significant challenge; however, several novel JAK inhibitors hold considerable promise for future treatment, such as pacritinib, momelotinib, and itacitinib [80, 90–92]. Ruxolitinib-based combinations are continuously increasing and have gradually improved according to clinical needs. There is a wide range of possibilities in research into promoting apoptosis, improving the hematopoietic stem cell microenvironment, TP53 signaling pathway, and telomerase inhibitors [93].

Some MF patients could experience severe thrombocytopenia after using ruxolitinib alone. Ruxolitinib-associated thrombocytopenia and anemia can be managed by dose adjustment, treatment discontinuation, or plus other drugs such as danazol, thalidomide, and lenalidomide. A run-in phase with ruxolitinib for 3 months followed by cautious introduction and gradual escalation of other drugs could improve the tolerability and efficacy. Ruxolitinib monotherapy is difficult to reduce WBC and PLT within normal ranges (WBC ≤ 10.0 × 10^9^/L or PLT ≤ 400 × 10^9^/L) [94, 95]. Hydroxyurea is commonly used for the control of thrombocytosis and leukocytosis [96]. Ruxolitinib combined with hydroxyurea need to be more standardized, especially in preventing “rebound” after hydroxyurea abrupt interruption. In another trial of ruxolitinib combination with hydroxyurea, the dose for hydroxyurea was chosen by clinician choice based on WBC and PLT.

Ruxolitinib before HSCT represents a new treatment strategy. HSCT has been used widely as a method to cure MF in recent years. In most clinical trials, ruxolitinib-induced hematological AEs would result in changed treatment strategies, such as dose adjustment, transfusion, or even dose discontinuation. Ruxolitinib combined with danazol or ESAs is effective in improving AR. The results are shown in Table 2. From the above trials, ruxolitinib combined with danazol or lenalidomide or azacytidine had certain advantages in improving JAK2/V617F allele burden and BM fibrosis. The results are shown in Table 2.

## Conclusions

Currently, except HSCT, all available treatments for MF are palliative and have limited impact on survival. The best practice administration of MF patients involves considering disease progression, age, comorbidities, and AEs. In addition, according to the patient’s physical condition and disease progress, a safe and effective treatment strategy should be formulated at the beginning of treatment, which should include evaluation of the pros and cons and prognosis of the present treatment strategies, including timely adjustment. The scientific study of the combination of different drugs, dosages, order of administration, and cycle of medication can bring more benefits to patients. With a large amount of research in molecular genetics and molecular biology, the pathogenesis of MF will become increasingly clear, which will lead to the development of new targeted drugs, and ultimately, a successful cure. Ruxolitinib combined with androgens, immunomodulatory agents, HDACi, ESAs, azacytidine, and hydroxyurea are currently being used in clinical treatment. The results of different combination trials demonstrate the superiority of the combinations over monotherapy trials in improving anemia, reducing spleen size, AEs, and prolonging OS; however, the safety and tolerability of the combination therapy frequently interferes with the continuation of trials, prompting the exploration of new drugs and new therapeutic targets.

Ruxolitinib before HSCT can improve the rate of successful transplantation, and ruxolitinib combined with danazol or ESAs showed excellent tolerability and safety. However, ruxolitinib combined with immunomodulatory agents requires additional drugs to improve tolerance and safety. The therapeutic effect of improving anemia and spleen response is prominent in ruxolitinib-based combinations. Gene mutation testing is crucial, particularly for patients with *JAK2* gene mutations, who might benefit more when receiving treatment using ruxolitinib-based combinations. As the disease progresses, drug doses need to be modified in some ruxolitinib-based combinations. Dose modification can affect efficacy, especially in terms of tolerance and adverse reactions. Considering the tolerance of patients with MF means that treatment can start with a low dose. Ruxolitinib-based combinations are a new clinical treatment strategy, and the results of some ruxolitinib-based combinations are partly encouraging.

With the continuous research of genomics, precision-based medicine has made significant progress. One of the biomarker-guided trials is umbrella trials, in which multiple targeted therapies are evaluated for a single disease that is stratified into multiple subgroups based on different molecular or other predictive risk factors. As next-generation sequencing continues to develop, umbrella trials can provide more nuanced assignment in matched treatment. Focus on testing of personalized multiple driving mutations, biomarker, and adoption of appropriate endpoints (i.e., for MPN-SAF, MPN-10 and IWG-MRT response criteria) are important considerations for researchers when designing ruxolitinib-based combinations in the treatment of MF [95, 97]. Asynchronous introduction of combination drugs should be considered when in the designing of trials. For example, it will be better to learn about the difference of the effect, safety and tolerance of monotherapy and combinationdrug when adding a monotherapy phase before combination-drug phase [98, 99].

To provide better clinical guidance, comparisons of these randomized controlled trials with the trials of ruxolitinib alone are necessary. JAK inhibition has become a preferred method of MPN therapy, and future research should focus on JAK inhibition–based combinations and the development of new JAK inhibitors [100].
